# Novel Membrane-Based Electrochemical Sensor for Real-Time Bio-Applications

**DOI:** 10.3390/s141122128

**Published:** 2014-11-24

**Authors:** Fatima AlZahra'a Alatraktchi, Tanya Bakmand, Maria Dimaki, Winnie E. Svendsen

**Affiliations:** Department of Micro- and Nanotechnology, Technical University of Denmark, Ørsteds Plads, 2800 Kgs. Lyngby, Denmark; E-Mails: tanba@nanotech.dtu.dk (T.B.); madi@nanotech.dtu.dk (M.D.); Winnie.Svendsen@nanotech.dtu.dk (W.E.S.)

**Keywords:** membrane electrodes, electrochemical sensing, membrane-sensor, real-time monitoring, dopamine, PC12 cells

## Abstract

This article presents a novel membrane-based sensor for real-time electrochemical investigations of cellular- or tissue cultures. The membrane sensor enables recording of electrical signals from a cell culture without any signal dilution, thus avoiding loss of sensitivity. Moreover, the porosity of the membrane provides optimal culturing conditions similar to existing culturing techniques allowing more efficient nutrient uptake and molecule release. The patterned sensor electrodes were fabricated on a porous membrane by electron-beam evaporation. The electrochemical performance of the membrane electrodes was characterized by cyclic voltammetry and chronoamperometry, and the detection of synthetic dopamine was demonstrated down to a concentration of 3.1 pM. Furthermore, to present the membrane-sensor functionality the dopamine release from cultured PC12 cells was successfully measured. The PC12 cells culturing experiments showed that the membrane-sensor was suitable as a cell culturing substrate for bio-applications. Real-time measurements of dopamine exocytosis in cell cultures were performed, where the transmitter release was recorded at the point of release. The developed membrane-sensor provides a new functionality to the standard culturing methods, enabling sensitive continuous *in vitro* monitoring and closely mimicking the *in vivo* conditions.

## 1. Introduction

The development of electrochemical sensors is a rapidly growing and popular area [1,2]. In recent years electrochemical sensors have attracted great attention in chemical and biological studies due to the high sensitivity, simplicity and reliability of the sensors [2–4]. Electrochemical sensors are used to provide information about, e.g., selected DNA sequences, mutated genes associated with human diseases or neurotransmitters [5,6]. The progression made in this field promises simple and accurate platforms for patient diagnostics [5].

Apart from the sensitivity and simplicity of electrochemical sensors, they allow *in vitro* and *in vivo* detection of analytes. *In vitro* research aims at mimicking the *in vivo* conditions to improve the understanding of the processes inside the body. Therefore, enhancing the *in vitro* techniques of electrochemical sensing is essential to achieve tools capable of providing a more exact picture of what is happening in the microenvironment of cellular cultures.

Cellular behavior has been explored by several types of electrochemical sensors in previous research. Castillo and colleagues explored a graphene electrode modified with a new conjugate of peptide nanotubes and folic acid for the selective detection of human cancer cells over-expressing folate receptors [4]. Another common example of electrical cell activity is the release of neurotransmitters generated from neurons that can be recorded using electrochemical sensors. In a previous study a combined cell culture biosensing platform using vertically aligned self-assembled peptide nanofibers was used for the detection of dopamine from PC12 cells [7]. Also in [8] the authors describe the use of overoxidized polypyrrole electrodes for the same purpose. Several other examples exist, using metal or carbon electrodes arranged in various geometries to electrochemically monitor various analytes in a cell culture.

To fully reproduce the various environments that cells and tissues encounter during growth, microporous membranes are an important alternative to solid culturing substrates [9–11]. Culturing on solid substrates forces the cells to uptake nutrients and release waste and signaling molecules through their top side. In contrast, microporous membranes provide a surface that mimics *in vivo* conditions better than solid substrates by e.g. allowing exposure to nutrients and waste release also from beneath the cells [9]. The traditional analysis of cellular cultures does not effectively allow continuous measurements of biological behavior such as dopamine exocytosis. As an example, HPLC measurements require discontinuous sampling that excludes valuable information that can be provided by continuous monitoring.

This study discloses a membrane-based sensor, where gold (Au) and silver-chloride (AgCl) electrodes with a thickness of a few nanometers are deposited on top of a microporous membrane surface. The membrane sensor allows aqueous medium to penetrate the membrane from beneath and reach the upper layer where cells are cultured. This permits optimal cell growth conditions as seen in current membrane culturing applications [9–11]. Moreover, monitoring of the target analyte in the proximity of the cells without any dilution or diffusion of the signals provides a more thorough understanding of processes occurring inside a culture. The membrane sensor was functionalized by culturing of the cells on top and detection of dopamine exocytosis in PC12 cells was demonstrated with this system.

## 2. Experimental Section

### 2.1. Materials/Chemicals

A dopamine stock solution at 0.1 M was made from dopaminehydrochloride (H8502-10G, Sigma-Aldrich, Broendby, Denmark, 0.47 g) in PBS (P4244, Sigma-Aldrich, 25 mL). A coating solution of polyethyleneimine (PEI) (P-3143, Sigma Aldrich) diluted to a concentration of 50 μg/mL in PBS was prepared. A concentration of 3.1 × 10^5^ PC12 cells (CRL-1721, ATCC, Manassas, VA, USA), passage 2, was cultured on each membrane-sensor. Differentiation medium was made from DMEM/F12 (D0819, Sigma-Aldrich), heat inactivated horse serum (HS, H1138, Sigma), fetal bovine serum (FBS, F-2442, Sigma-Aldrich), penicillin/streptomycin (P/S), 4-(2-hydroxyethyl)-1-piperazineethanesulfonic acid (HEPES, 83264, Sigma-Aldrich), and Nerve Growth Factor (NGF, N2513, Sigma-Aldrich). Trypsin-EDTA (T2600000, Sigma-Aldrich) was used for release of cells prior to reseeding on the sensor.

### 2.2. Membrane-Sensor Design

**Figure 1.**
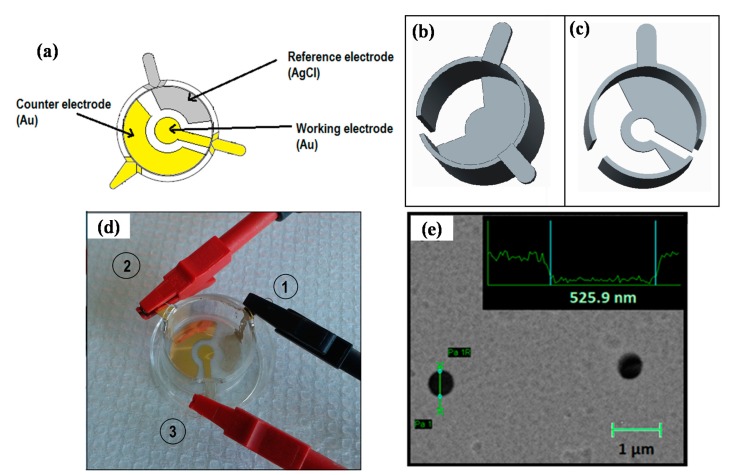
(**a**) Sketch of the three-electrode membrane-based electrochemical sensor. The sensor involves patterned deposition of a working, counter and reference electrode; (**b**) Sketch of the shadow mask used to deposit the reference electrode; (**c**) Sketch of the shadow mask used to deposit the working and the counter electrodes; (**d**) Membrane-sensors connected to a potentiostat by crocodile clips. (1) Reference electrode; (2) Counter electrode and (3) Working electrode; (**e**) SEM image of the electrode covered membrane showing black dots representing the pores. The inset shows a line scan across a single pore revealing that the pores were not blocked by the metal deposition.

The membrane sensor is based on the existing membrane inserts made of hydrophilic PTFE with a diameter of 24 mm and average pore size of 400 nm (PICM0RG50, Merck Millipore, Billerica, MA, USA). The three electrode membrane based electrochemical biosensor requires a working electrode, counter electrode and a reference electrode, see Figure 1a. The working and counter electrodes were made of Au, while the reference electrode was made of AgCl. The pattern of the electrodes satisfies the requirement of having a larger surface area of the counter electrode than the working electrode [12]. The pattern of the electrodes deposited on the membrane, with electrical contacts from the membrane to the sides of the grips, is seen in Figure 1a.

### 2.3. Shadow Masks and Patterned Electrode Deposition

In order to deposit the desired pattern, two different shadow masks were designed, as seen in Figure 1b,c. The shadow masks were milled in aluminum. Electron-beam evaporation was used to deposit metal on the membrane inserts using the pattern masks and a Physimeca electron beam evaporation system (Physimeca Technologies, Villiers Le Bacle, France). The deposition was executed in two steps: firstly by depositing the Ag-pattern (Figure 1b) and secondly by depositing the Au-pattern (Figure 1c). Ag was deposited with a rate of 5 Å/s, while Au was deposited with a rate of 10 Å/s, both at 80 °C and a pressure of 2 × 10^6^ mbar. In order to deposit material on the sides of the membrane insert the electron-beam was tilted 25°. A layer of 100 nm Ag and Au was deposited. The Ag on the membrane-sensors was covered with 50 mM iron(III)chloride-(FeCl_3_) for 12 s in order to create a film of approximately 50 nm AgCl [13].

### 2.4. Conductivity Measurements and Pore Characterization

The conductivity of the membrane sensor was measured using a two-point probe ohmmeter. The measurements were taken between the membrane-electrodes and their respective grips. It was necessary to add a small amount of silver paste (DGP, Advanced Nano Products, Seoul, Korea) in the corner between the wall and the membrane, since the connection was disrupted. By adding silver paste, the current could successfully be measured from the membrane to all grips. The pores of the membrane-sensor were characterized using a Scanning Electron Microscope (SEM, Zeiss Supra 40 VP, Cambridge, UK) in order to verify the porosity of the membrane.

### 2.5. Electrochemical Characterization

Cyclic voltammetry was used to characterize the performance of the membrane sensor, while chronoamperometry was used to determine the detection limit using synthetic dopamine as a model compound. The membrane sensor was connected to the potentiostat via alligator clips as seen in Figure 1d. Ferri/ferrocyanide (FFC) in phosphate buffered saline (PBS) was used to characterize the membrane sensor. Cyclic voltammograms were obtained in a 10 mM FFC aqueous solution, where the voltage was cycled from −0.6 to 0.8 V for several sweep rates between 50 and 260 mV/s, starting with the highest sweep rate.

Synthetic dopamine (as described in Section 2.1) was used to determine the lowest detectable concentration by the membrane sensor. The stock solution was purged with nitrogen for 30 min before starting the experiments to eliminate oxygen or any other form of oxidation during the process [14]. All electrochemical characterization was performed in PBS. The tests were performed at room temperature. The potential used for the chronoamperometry measurements was 200 mV based on the dopamine peak found during the CVs. Prior to injecting the dopamine a baseline was obtained in PBS for 60 s. Then, the dopamine solution was pipetted at the edge of the membrane and the current was monitored for about 3 min, until it reached the baseline again. In this work a new membrane was used for each concentration of synthetic dopamine. However, it is possible to perform repetitive measurements with the same membrane as is demonstrated in a later developed system [15].

### 2.6. PC12 Cells Culturing on Membrane-Sensor

Prior to seeding of the cells the membranes were sterilized by washing in ethanol. In order to promote cellular adhesion the membrane sensors were coated with a PEI layer. The membranes were submerged in 1 mL of 50 μg/mL PEI in PBS for two hours at room temperature. Subsequently, the sensors were washed twice with PBS before cell culturing.

The PC12 cell culturing was conducted in differentiation medium according to the procedure followed by Taskin and others [7]. Briefly, 24 h before seeding the PC12 cells on the membrane sensors the cell culture medium was changed to differentiation medium consisting of DMEM/F12 supplemented with 0.5% HS, 0.5% FBS, 100 μg/mL P/S, 25 mM HEPES, and 0.1 μg/mL NGF. This served to initiate the differentiation and inhibit proliferation.

After 24 h of pre-differentiation the differentiation medium was removed from the culture flask and the cells were washed 3 times with PBS. The cells were detached by incubation with 1 mL 0.05% trypsin-EDTA at 37 °C for 3 min. After the incubation the cell suspension was transferred to a 10 mL Falcon tube and 4 mL of differentiation medium was added to passivate the trypsin-EDTA. Next, the tube was centrifuged at 850 rpm for 3 min. The supernatant was removed and the cell pellet was re-suspended in differentiation medium. The cells were then seeded on the membrane sensors with a surface density of 0.7 × 10^5^ cells/cm^2^, corresponding to 3.1 × 10^5^ cells per membrane. The membrane sensors with cells were transferred to 6-well plates containing 2 mL of differentiation medium per well. The well plates were kept in a humidified incubator at 37 °C, where the cells were cultured for either 14 or 30 days with a medium change every 2–3 days.

The PC12 cells that were used for measuring dopamine exocytosis were cultured on the membrane sensors for 14 days in five replicates before the dopamine release was measured from each culture through chronoamperometry.

Prior to exocytosis measurements the cell medium was removed from the well plates and replaced by 5 mM physiological buffer solution (low K^+^ buffer) using Eppendorf pipettes. A constant potential of 200 mV was applied and the current was measured, producing a baseline. When a stable baseline was obtained dopamine release was triggered by injecting 200 μL of 450 mM potassium buffer solution (high K^+^ buffer) directly on top of the cells [11].

## 3. Results and Discussion

### 3.1. Confirming the Porosity of the Membrane-Sensor

SEM analysis was performed to determine the state of the pores after the metal deposition. The obtained images of the patterned electrodes confirmed that the pores were not blocked by the deposited materials (Figure 1e). Line scans of 30 pores show an average pore diameter of 521 ± 29 nm (Figure 1e, inset). This shows that deposition of 100 nm thick electrodes does not compromise the porosity of the membrane-sensor.

### 3.2. Evaluation of the Electrochemical Performance of the Membrane-Sensor

The membrane sensor showed good conductivity between the patterned electrodes and the grips with a resistance between 6.4 and 13.1 Ω, see Table 1 for average results measured on five different electrodes.

**Figure 2.**
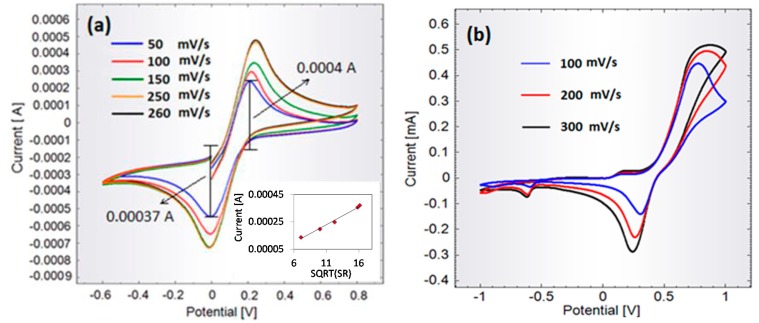
Cyclic voltammograms obtained with membrane-sensor. (**a**) Typical cyclic voltammograms in 10 mM Ferricyanide at differential potential scan rates. Inset: Current peak heights *versus* square root of the sweep rates (SR); (**b**) Examples of typical cyclic voltammograms in 0.1 M synthetic dopamine at various potential scan rates.

Evaluation of the cyclic voltammetry measurements with different potential sweep rates in FFC leads to the conclusion that the membrane sensor is reliable, Figure 2a.

It is seen that the numeric ratio between the current peaks is almost unity (example in figure: 0.0004 A/0.00037 A) and that the peak potentials are independent of the sweep rate. The inset in Figure 2a shows that the peak current is proportional to the square root of the sweep rate. Based on these observations, the reaction on the membrane-sensor is considered pseudo-reversible.

The electrochemical response of the membrane sensor to 0.1 M synthetic dopamine (in PBS) clearly demonstrates oxidation (top anodic peaks) and reduction (bottom cathodic peaks) in the forward and reversed scans, Figure 2b. Consequently the measurements indicate the expected reversibility of the reaction obtained by the membrane sensor.

**Figure 3.**
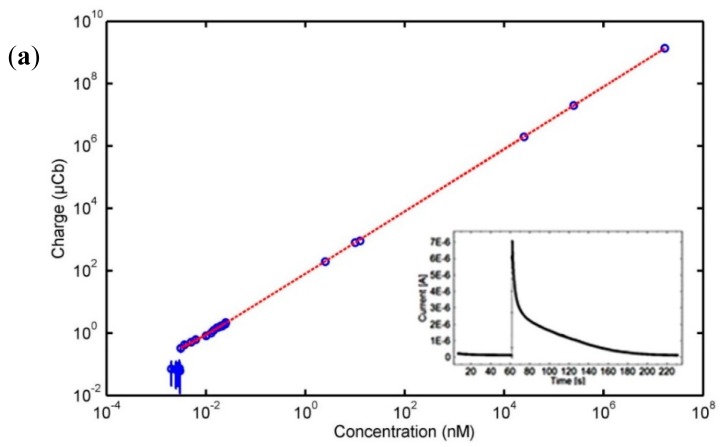

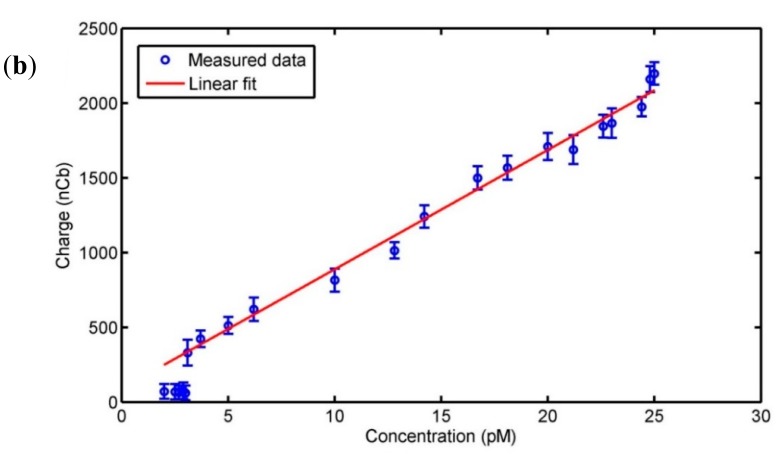
Calibration of membrane-sensor. Plot of the average charge signals *versus* dopamine concentrations for chronoamperometric responses obtained by the membrane-sensor. Error bars denote standard deviation of the measurements. (**a**) The measurements obtained for the entire range of tested concentrations; (**b**) The measurements obtained at the lower concentration range (up to 25 pM). Inset: Typical chronoamperometric recording of dopamine.

Chronoamperometric measurements were used to produce a calibration curve for detection of dopamine using the membrane sensor. The measurements were conducted by applying a constant potential of 200 mV and measuring the current response. The potential was chosen based on the position of the oxidation peak in Figure 2b. Each measurement was initiated in PBS and once a stable baseline was obtained a controlled amount of dopamine was added. The inset in Figure 3 shows a typical chronoamperometric time-trace generated upon injection of dopamine solution. The accumulated charge (area beneath the curve [16]) was calculated using numerical integration by the trapezoidal rule. Figure 3a shows the accumulated charge of the amperometric measurements plotted against dopamine concentration. It is seen that the current signal increases linearly with dopamine concentration for concentrations varying from 3.1 pM to 17 mM. The linear fit (shown with the solid red line in Figure 3a following the equation *Charger* (*μCb*) = 79.82 · *Concentration* (*nM*) + 0.09, with an R^2^ value of 0.99) was based on the data obtained for concentrations up to 25 pM, as these measurements were repeated several times (Figure 3b). The measurements for higher concentrations of dopamine were only done once and this is why this data has not been used to obtain the linear fit. However, by extending the fit to the higher concentrations (shown with the dotted red line in Figure 3a), we observe that these also fall on the fitted line. Therefore, we can conclude that linearity is observed up to mM range.

In the horizontal range of the first five points in Figure 3b, the increase of dopamine concentration did not lead to an increase in signal. Thus, 3.1 pM was the lowest detected dopamine concentration with the membrane sensor presented in this study.

In this work the effect of interfering compounds, such as e.g. ascorbic acid, has not been addressed. However, it is a commonly encountered problem when utilizing amperometric sensing and thus it should be mentioned that numerous methods to avoid or diminish the issue have previously been demonstrated [17–22]. As the developed membrane sensor has electrodes made by commonly used metals, it is likely that the methods developed for other systems can be adapted to this system with good results. This will be addressed in future work along with demonstrations on sensing in sample fluids such as saliva, blood or urine.

### 3.3. PC12 Cell Characterization on the Membrane-Sensor

**Figure 4.**
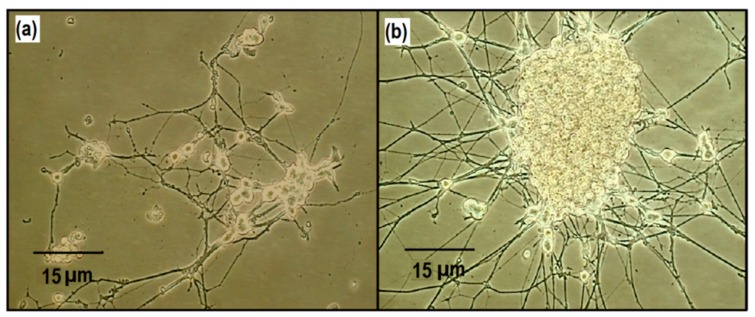
PC12 cells differentiated on the membrane-sensor. Differentiation for (**a**) 14 days and (**b**) 30 days. The characteristic neuronal-like morphology is exhibited by the cells.

To investigate the possibility of cellular studies on the membrane sensor, PC12 cells were cultured on top of the sensor surface. Figure 4a displays the healthy differentiation of the cells on top of the membrane sensor after 14 days of culturing.

Although PC12 cells are normally cultured for a maximum of about 14 days [23], the PC12 cells were also cultured on the membrane sensor for 30 days likewise with healthy differentiation (Figure 4b). The differentiation into the characteristic neuronal cell morphology is clearly observed, which is indicative for healthy survival and development. This demonstrates that cells cultured on the membrane sensor were capable of proper growth and differentiation.

### 3.4. Real-Time Dopamine Exocytosis Measurements of PC12 Cells on Membrane-Sensor

**Figure 5.**
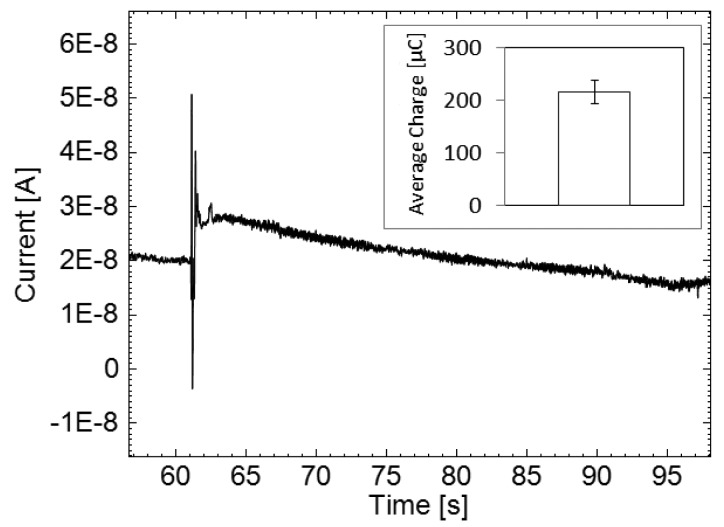
Typical chronoamperometric current-time trace corresponding to dopamine exocytosis from differentiated PC12 cells, obtained upon induction of dopamine release on top of the membrane-sensor. Inset shows the average charge accumulated during the measurement.

Real-time dopamine exocytosis measurements were obtained using the membrane-sensor (Figure 5). The dopamine release was triggered approximately 60 s after recording a baseline in pure PBS, and the dopamine was measured. The inset in Figure 5 shows an average charge recording of ∼216 μC released by an average cell population of 3.1 × 10^5^ per membrane-sensor for five experiments. According to the calibration curve in Figure 3 and the uncertainties of the linear fit, approximately 2.71 ± 0.16 nM dopamine was detected by one membrane sensor. This corresponds to an average generated charge of 0.7 nC/cell. The generated charge per PC12 cell measured by the membrane sensor is comparable to an earlier study that detected 0.1 nC/cell using gold-deposited electrodes [24].

The results show that it is possible to obtain recordings of neurotransmission as the signals are released, which makes the membrane-sensor suitable for monitoring purposes. The membrane-sensor would be advantageous to use in bio-applications, where small changes need to be detected in conditions mimicking the *in vivo* environment, because of its high sensitivity and low detection limit compared to traditional detection methods with detection limits in the nanomolar range. The sensor competes with single-walled carbon nanotubes decorated sensors with a detection limit in the picomolar range [1,25–27].

In this work only one measurement per membrane was performed. However, a similar sensor system has later been proven to be capable of continuous measurements as well as repeated measurements over a longer time period [15]. This indicates that the sensor presented in this work has potential as a reusable sensor system, which would make it suitable for integration with microfluidics.

## 4. Conclusions

A novel membrane-based sensor has been presented, where patterned electrodes have been deposited on a thin porous membrane. The membrane sensor allows continuous real-time measurements of analytes of interest from cell cultures without dilution of target signals. Furthermore, electrochemical characterization of the sensor has shown reliability of measurements and concentrations of dopamine in the picomolar range have successfully been detected. The application of the membrane sensor in culturing PC12 cells and detecting released dopamine from the cell populations demonstrates that this novel sensor can with great advantages be integrated in future biological studies.

## Figures and Tables

**Table 1.** Electrode resistances of the membrane-sensor.

**Electrode**	**Resistance**
Counter	6.4 ± 0.2 Ω
Working	7.2 ± 0.5 Ω
Reference	13.1 ± 0.6 Ω
